# CycPeptMP: enhancing membrane permeability prediction of cyclic peptides with multi-level molecular features and data augmentation

**DOI:** 10.1093/bib/bbae417

**Published:** 2024-08-29

**Authors:** Jianan Li, Keisuke Yanagisawa, Yutaka Akiyama

**Affiliations:** Department of Computer Science, School of Computing, Tokyo Institute of Technology, Tokyo 1528550, Japan; Department of Computer Science, School of Computing, Tokyo Institute of Technology, Tokyo 1528550, Japan; Middle-Molecule ITbased Drug Discovery Laboratory (MIDL), Tokyo Institute of Technology, Tokyo 1528550, Japan; Department of Computer Science, School of Computing, Tokyo Institute of Technology, Tokyo 1528550, Japan; Middle-Molecule ITbased Drug Discovery Laboratory (MIDL), Tokyo Institute of Technology, Tokyo 1528550, Japan

**Keywords:** cyclic peptide, deep learning, data augmentation, feature design, membrane permeability

## Abstract

Cyclic peptides are versatile therapeutic agents that boast high binding affinity, minimal toxicity, and the potential to engage challenging protein targets. However, the pharmaceutical utility of cyclic peptides is limited by their low membrane permeability—an essential indicator of oral bioavailability and intracellular targeting. Current machine learning-based models of cyclic peptide permeability show variable performance owing to the limitations of experimental data. Furthermore, these methods use features derived from the whole molecule that have traditionally been used to predict small molecules and ignore the unique structural properties of cyclic peptides. This study presents CycPeptMP: an accurate and efficient method to predict cyclic peptide membrane permeability. We designed features for cyclic peptides at the atom-, monomer-, and peptide-levels and seamlessly integrated these into a fusion model using deep learning technology. Additionally, we applied various data augmentation techniques to enhance model training efficiency using the latest data. The fusion model exhibited excellent prediction performance for the logarithm of permeability, with a mean absolute error of $0.355$ and correlation coefficient of $0.883$. Ablation studies demonstrated that all feature levels contributed and were relatively essential to predicting membrane permeability, confirming the effectiveness of augmentation to improve prediction accuracy. A comparison with a molecular dynamics-based method showed that CycPeptMP accurately predicted peptide permeability, which is otherwise difficult to predict using simulations.

## Introduction

Insulin was the first synthesized therapeutic peptide in $1921$. Subsequently, peptides were frequently studied as they combine the advantages of small molecule and antibody drugs. The growing advancements in genetic engineering, peptide synthesis technologies, and sequence analysis tools have led to the development of new classes of peptide therapeutics for various applications [1–4]. Meanwhile, many computational methods have been developed to efficiently predict the properties of linear peptides [5–7]. However, certain limitations of conventional linear peptides, such as low stability, selectivity, and cell membrane permeability remain unresolved [8, 9]. In contrast to linear peptides, the unique structural features of macrocyclic peptides stem from their restricted conformational flexibility and local secondary structure motifs, allowing for bioactive conformations with remarkable potency and selectivity [10, 11]. Therefore, the value of cyclic peptides is increasing in pharmaceutical research due to their high binding affinity, stability, target selectivity, and ability to inhibit intracellular protein–protein interactions [12–14]. In the past century, cyclic peptide drugs were predominantly sourced from natural products, including antimicrobial agents and human peptide hormones. Recent advances in novel synthesis and screening systems have led to breakthroughs in cyclic peptide drug discovery [15, 16]. For example, the random nonstandard peptides integrated discovery (RaPID) system designs cyclic peptides from a diverse library, including non-natural amino acids, enabling the synthesis and rapid selection of potent binders for a wide range of therapeutic targets [17]. The RaPID system has designed novel cyclic peptides for complex therapeutic targets, including a high-affinity binder to the osteoporosis target PlexinB1 [18], an inhibitor of the ubiquitin-protein ligase E6AP [17], and a selective inhibitor of the oncogenic K-Ras [19]. Over $40$ cyclic peptide drugs are currently in clinical use, with the FDA having approved approximately one macrocyclic peptide drug per year for the past $20$ years [16, 20]. Despite their pharmacological potential, cyclic peptides often exhibit poor membrane permeability, severely limiting their biological applications and development of orally available drugs [9]. The mechanism underlying membrane permeation by cyclic peptides remains unclear; however, cyclic peptides with a “closed”-conformation in hydrophobic environments often exhibit enhanced permeability [21, 22]. The “closed”-conformation conceals polar groups through intramolecular hydrogen bonds and lipophilic side chains, contributing to their increased permeation efficiency. Drawing inspiration from the structure of cyclosporin A, a naturally occurring N-methylated macrocyclic peptide with high permeability, shielding of the exposed hydrogen bond donor (-NH) through N-methylation has been widely employed to enhance membrane permeability [23, 24]. Various strategies, such as conformational control [12, 25], amide-to-ester substitution [26], amide-to-thioamide substitution [10], and side-chain modifications [27] have emerged for improving membrane permeability. However, these strategies do not improve membrane permeability across all cyclic peptides.

Selection of candidate compounds with high membrane permeability is important during the early stages of drug development. Thus, due to the cost associated with randomly measuring the permeability of numerous peptides using biochemical assays, the development of a rapid computational method that enables the prediction of membrane permeability is eagerly anticipated. Computational approaches to predict the permeability of cyclic peptides have been primarily based on molecular dynamics (MD) simulation [28–31]. Markov state models were used to analyse simulation data and elucidate cyclic peptide behavior [32], which is crucial to understanding membrane permeation mechanisms and optimizing structures to enhance membrane permeability. However, the computational cost of MD-based methods is a major limitation. In contrast to MD-based methods, several physicochemical or machine learning models have been developed, offering more rapid prediction capabilities [33–37]. The descriptors for hydrophobicity, such as the octanol-water partition coefficient (LogP), are generally the most important features for prediction. However, unlike the property prediction methods for small molecules and linear peptides, which have considerable experimental data, these models were established using limited data sets ($10$–$250$ cyclic peptides) and, thus, lack a sufficient degree of generalization performance. Furthermore, these methods directly apply whole-molecule features, typically used to predict the membrane permeability of small molecules while ignoring the unique structural characteristics of cyclic peptides, such as sequence information and circularity.

Unlike conventional physicochemical and machine learning approaches, deep learning (DL) models offer an architectural design tailored to peptide characteristics [38] and can automatically extract more complex structural features than small-molecule compounds from datasets. DL-based small molecule property prediction methods based on graph neural networks (GNNs) and transformers have become a major research area. By representing atoms as nodes and bonds as edges, the GNN-based method can effectively capture molecular structural information and integrate the topological structure of molecules with complex atomic features. Nonetheless, most existing approaches, such as GCN [39, 40], GAT [41, 42], and MPNN [43] have intrinsic limitations, including a poor ability to process global information and risk of over-smoothing when many atoms are present. In contrast, many transformer-based methods have been proposed that treat SMILES as strings following the successful experience in natural language processing [44–46]. Since these methods lack structural information, several methods have been developed using molecular graph representations as input that can encode more complex atom and bond information than strings [47–50]. However, cyclic peptide permeability datasets are limited and diverse, with discrepancies and errors stemming from the use of various assay systems. To address these limitations, a comprehensive database of cyclic peptide membrane permeability was constructed, called CycPeptMPDB [51]. CycPeptMPDB comprises information on $7334$ cyclic peptides, including structures and experimentally measured membrane permeabilities, from $45$ published studies and $2$ patents from pharmaceutical companies. It represents the first platform for developing DL-based prediction methods. Interestingly, over $99.6\%$ of cyclic peptides include non-natural amino acids, suggesting that they were created to enhance permeability through chemical modifications, such as N-methylation, or by deliberately incorporating non-natural building blocks in their design. In addition to the experimental data, the database contains relevant supporting information, such as 3D conformations and hierarchical editing language for macromolecules (HELM) sequence representations, which are composed of uniquely defined monomers (substructures, such as residues). This allows users to analyse data for various applications.

Cyclic peptides are characterized by complex conformational dynamics, where even a minor alteration in a single residue can lead to substantial changes in their membrane permeability [52]. Therefore, many publications in CycPeptMPDB have focused on measuring changes in membrane permeability while only varying a few residues and maintaining a largely constant sequence. The combined use of multi-scale molecular features can improve the accuracy of predicting small molecule properties [49, 53] and peptide–protein binding [54]. This study proposes CycPeptMP: a membrane permeability prediction model for cyclic peptides that effectively integrates multi-level features with state-of-the-art DL techniques. We engineered features at the atom, monomer, and peptide levels to concurrently capture the local sequence variations and global conformational changes in cyclic peptides. Additionally, we employed data augmentation methods at the atom, monomer, and peptide levels to enhance the training efficiency of our model for complex cyclic peptides.

## Materials and Methods

### Experimental dataset

We used the structure and logarithm of experimentally determined membrane permeability ($\mathrm{LogP_{exp}}$) of peptides in CycPeptMPDB. CycPeptMPDB contains permeability data based on the parallel artificial membrane permeability (PAMPA), Caco-2, Madin-Darby canine kidney (MDCK), and Ralph Russ canine kidney (RRCK) assays. We selected PAMPA entries with the largest number of data points. The value recorded in the latest publication was used if the same peptide was measured in multiple publications. Consequently, $6889$ peptides were selected, covering a relatively wide range of molecular weights, from $342.44$ to $1777.74$. Considering that the lower limit of $\mathrm{LogP_{exp}}$ in CycPeptMPDB was $-10$ ($1\times 10^{-10}\text{cm/s}$, $240$ peptides), but the detection limit in most publications was $-8$ ($1\times 10^{-8}\text{cm/s}$), we rounded the permeabilities of $314$ peptides with values lower than $-8$ to $-8$. Similarly, the permeability of one peptide with a value higher than $-4$ was rounded to $-4$.

**Figure 1:**
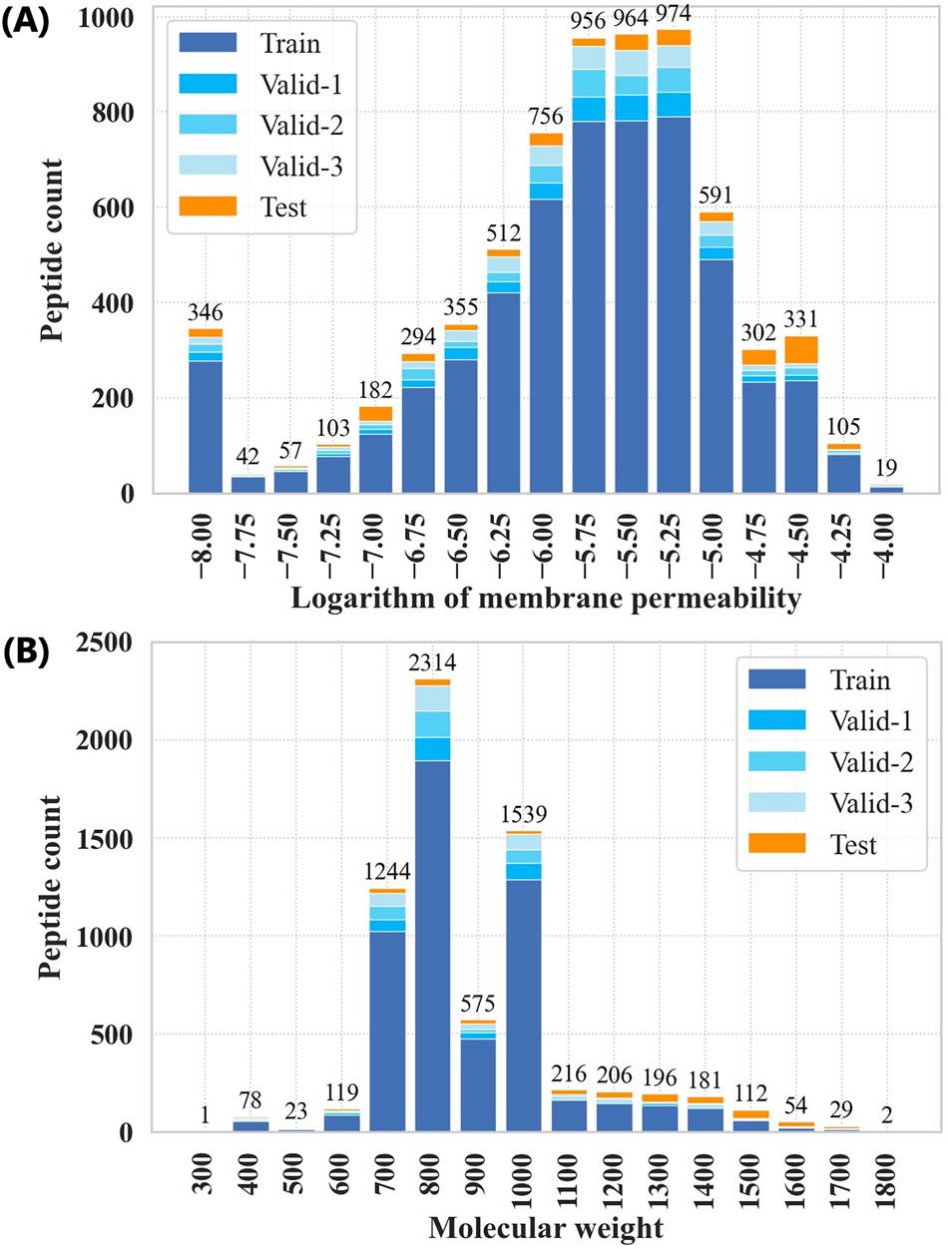
Experimental data distribution. (A) Logarithm of experimentally determined membrane permeability ($\mathrm{LogP_{exp}}$). (B) Molecular weight. Valid–1 is the dataset used for the first-time evaluation of the validation set; the corresponding training data sets are Train, Valid–2, and Valid–3.

The validation and test sets were extracted from the overall data for model evaluation. First, the Kennard–Stone (KS) algorithm was employed to extract $5\%$ of all data ($344$ peptides) as the test set, which should uniformly cover the multidimensional space [55]. We generated $2048$-bit Morgan fingerprints (Morgan FP, radius: $2$) and selected the test set so that the Euclidean distance between each data point was maximized by the KS algorithm. From the remaining data, we randomly extracted $5\%$ validation sets ($344$ peptides) three times for parameter tuning, with no overlap between the three datasets. The membrane permeability and molecular weight distributions for each set are shown in Fig. 1. The average mean absolute error (MAE), mean squared error (MSE), correlation coefficient (R), and coefficient of determination ($\mathrm{R^{2}}$) from three repeated runs were used as evaluation metrics.

### Overview of CycPeptMP framework

**Figure 2:**
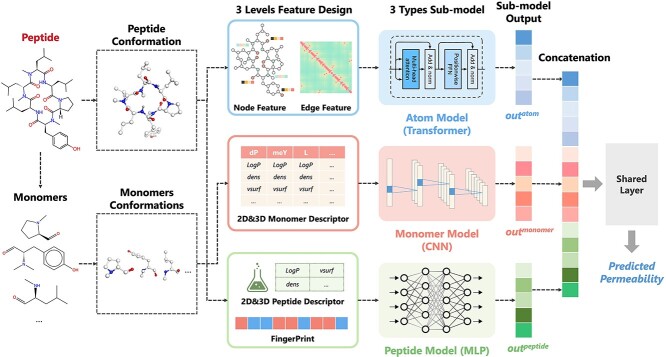
Overall framework of the CycPeptMP model. The model incorporates the transformer-based atom, convolutional neural network (CNN)-based monomer, and MLP-based peptide sub-models. The three-level expression vectors extracted using the three sub-models are concatenated and passed through a shared layer to derive the final permeability prediction value.

Figure 2 shows the overall architecture of the CycPeptMP model. We designed three-level representations of peptides and used each for three different sub-models to extract the atom-, monomer-, and peptide-level molecular representations. Initially, the input peptide was divided into monomers, and respective 3D conformations were generated from the peptide and monomers. Subsequently, atom- and peptide-level features were extracted from the peptide conformation and used as input for the atom and peptide models, respectively. Monomer-level features extracted from the monomer conformation were used as inputs for the monomer model. Finally, the three-level latent feature vectors extracted using the three sub-models were combined to derive the membrane permeability prediction values.

### Division of the monomer

Unlike small molecules, most cyclic peptides are composed of a combination of monomers that are the standard building blocks in their chemical synthesis. CycPeptMPDB also provides a monomer-level sequence representation because most membrane permeability studies of cyclic peptides performed modifications at the monomer level. Therefore, we designed monomer-level features to accurately capture the subtle sequence variations of cyclic peptides. The adopted definition of a monomer corresponded to that provided by CycPeptMPDB. Although the peptide and ester bonds on the side chain were cleaved in CycPeptMPDB, bonds existing anywhere other than the macrocycle were not subjected to division to fully express the properties of the local structure. Merely hydrolysing the peptide bond could generate a new hydrogen bond donor, potentially misrepresenting the substructure’s original physicochemical properties. Hence, an appropriate capping is required when decomposing peptides into monomers. When generating the conformation and calculating the monomer descriptor, the cleaved amide group or O atom of the amide-to-ester substitution was methylated (addition of CH3), and the carboxyl group was converted to an aldehyde (addition of H).

### Peptide and monomer descriptors

To design peptide- and monomer-level features, a whole peptide and each of its monomers were represented by $16$ descriptors, respectively, including LogP and polar surface area (Table 1; the correlation matrix of the selected descriptors is shown in Supplementary Fig. S1). Initially, the 3D conformations of peptides and monomers were generated using the RDKit package (version 2022.09.5) [56]. The initial structures were generated using the ETKDG method and then optimized by energy minimization with the UFF force field. Subsequently, the 2D and 3D descriptors were calculated using a single-conformation 3D structure. The $16$ descriptors were selected as follows: first, a total of $1857$ descriptors ($1689$ 2D and $168$ 3D descriptors) were calculated for whole cyclic peptides (peptide descriptors) and all monomers (monomer descriptors) using MOE software (version 2019.01) [57], the Mordred package (version 1.2) [58], and RDKit package. For 2D and 3D peptide descriptors, we removed all descriptors with constant values among cyclic peptides within the dataset. For descriptor pairs with an absolute correlation coefficient of $0.9$ or more, the one with the lower correlation with permeability was excluded. Consequently, $407$ ($335$ 2D and $72$ 3D descriptors) peptide descriptors were selected. Performing further feature selection and using only important features can reduce overfitting and improve model interpretability. Random forest (RF) is commonly used as an algorithm for robust feature selections, even with many variables. It can provide quantitative measures of the importance of each variable in prediction. Therefore, we constructed two RF models with the 2D or 3D peptide descriptors. Subsequently, seven 2D and nine 3D peptide descriptors were selected based on the assigned feature importance (Supplementary Fig. S2). Finally, the same $16$ monomer descriptors were selected, and peptide and monomer descriptors were standardized based on the Z-score.

**Table 1:** Selected descriptors, arranged in order of importance

Type	Name	Calculated by	Description
2D	Vsa:EState9	Mordred	Van der Waals surface area using EState indices and surface area contribution
	density	MOE	Molecular mass density (molecular weight divided by approximated van der Waals volume)
	MolLogP	RDKit	Wildman–Crippen LogP value
	fr_Al_OH	RDKit	Number of aliphatic hydroxyl groups
	logP(o/w)	MOE	Log of the octanol/water partition coefficient
	lip_violation	MOE	Number of violations of Lipinski’s Rule of Five
	h_logD	MOE	Log of the octanol/water distribution coefficient at pH 7
3D	dens	MOE	Molecular mass density (molecular weight divided by 3D van der Waals volume)
	FNSA4	Mordred	Fractional charged partial negative surface area (version 4)
	RNCS	Mordred	Relative negative charge surface area
	FASA-	MOE	Fractional water accessible surface area of all atoms with negative partial charge
	FCASA+	MOE	Fractional positive charge weighted surface area
	FAsa:P	MOE	Fractional water accessible surface area of all polar atoms
	FNSA2	Mordred	Fractional charged partial negative surface area (version 2)
	FNSA5	Mordred	Fractional charged partial negative surface area (version 5)
	vsurf_Wp2	MOE	VolSurf polar volume

### Atom model

We designed atom-level features so that the atom model could capture minor changes, such as enantiomers, by node features ($Node$) and global changes in the entire molecule by three types of node-pair relative relationship matrices ($Bond$, $Graph$, and $Conf$). As shown in Table 2, heavy atoms were considered nodes, and node features were represented as $Node$, bonded interaction weights were represented as $Bond \in \mathbb{R}^{N_{\mathit{atoms}} \times N_{\mathit{atoms}}}$, graphic pairwise distances were represented as $Graph \in \mathbb{R}^{N_{\mathit{atoms}} \times N_{\mathit{atoms}}}$, and 3D pairwise distances were represented as $Conf \in \mathbb{R}^{N_{\mathit{atoms}} \times N_{\mathit{atoms}}}$.

**Table 2:** Atom model input features

Type	Feature	Size	Description
Atom	Atom type	8	Type of atom by atomic number (one-hot)
	Degree of atom	5	Number of bonds (one-hot)
	Number of hydrogen	5	Number of bonded hydrogen atoms (one-hot)
	Formal charge	4	Electrical charge (one-hot)
	Hybridization	3	Type of atom hybridization (one-hot)
	Chirality	3	Type of atom chirality (one-hot)
	Is in a ring	1	Whether included in a ring structure
	Is aromatic	1	Whether aromatic
Bond	Bond type ($Bond$)	1	Single(1.0), Double(2.0), Triple(3.0), Aromatic(1.5), Conjugated(1.4), and No-bond (0)
Distance	Graph distance ($Graph$)	1	Distance calculated from graph representation
	3D distance ($Conf$)	1	Euclidean distance (Å) calculated from 3D conformation

**Figure 3:**
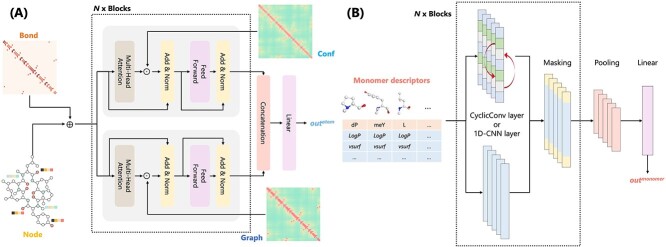
(A) Atom model architecture. Node features and three types of node-pair relative relationship matrices were used as input for the transformer-based model. (B) Architecture of the monomer model. Monomer descriptors were aligned based on the sequence information and used as input for the CNN-based model.

Since the transformer can effectively learn relationships between distant atoms even when the number of atoms is large (the maximum number of heavy atoms in the experimental data was $128$) [59], we constructed a transformer-based atom model to capture the overall graph structure and 3D conformation of the peptide (Fig. 3 (A)). $Bond$ recorded the molecule bond information and controlled message propagation between neighboring nodes by assigning weights to each bond type. According to the chemical bonding principle, bonds with more electron participation (such as unsaturated bonds) were assigned higher weights to enhance the exchange of information between atoms [48]. Meanwhile, many transformer-based models record the positional relationships between nodes or tokens using traditional absolute positional encoding [45, 60]. However, some studies have reported that relative positional encoding can improve prediction accuracy [47, 59]. To capture the local relationship between each node, embedded $Bond$ was used for positional encoding and added to embedded $Node$ to serve as the input for the encoder block as follows: 


(1) \begin{align*}& x = \frac{W_{\mathit{node}} Node + W_{\mathit{bond}} Bond}{\sqrt{d_{\mathit{model}}}},\end{align*}

where $d_{\mathit{model}}$ is the attention dimension of the atom model, $W_{\mathit{node}} \in \mathbb{R}^{N_{\mathit{node-features}} \times d_{\mathit{model}}}$ and $W_{\mathit{bond}} \in \mathbb{R}^{N_{\mathit{atoms}} \times d_{\mathit{model}}}$ are trainable parameters, and $x \in \mathbb{R}^{N_{\mathit{atoms}} \times d_{\mathit{model}}}$ is the updated input for the encoder block.

Two types of distance matrices, $Graph$ and $Conf$, i.e. the shortest pairwise graph distance and 3D Euclidean distance of each atom, were used to capture the peptide’s overall structure and 3D conformation. The distance maps were processed through an attenuation function to weaken distant interactions as follows: 


(2a) \begin{align*} Strength^{\mathit{graph}}_{i, j} &= \left\{ \begin{array}{@{}cc} 1 & (i = j) \\ \frac{1}{\mathit{Graph}_{i, j}} & (i \ne j) \end{array} \right.\!\!, \end{align*}

(2b) \begin{align*} Strength^{\mathit{conf}}_{i, j} &= \left\{ \begin{array}{@{}cc} 1 & (i = j) \\ \frac{1}{\mathit{Conf}_{i, j}} & (i \ne j) \end{array} \right.\!\!, \end{align*}

where $\mathit{Graph}_{i, j}$ and $\mathit{Conf}_{i, j}$ are the distance calculated between atom pairs $i$ and $j$ from the graph representation and 3D conformation. Furthermore, we designed a structure-enhanced transformer encoder to learn the structural and 3D conformational information of peptides using focused attention. The encoder comprised two blocks, one using $Strength^{\mathit{graph}}$ and another using $Strength^{\mathit{conf}}$. This approach attenuates attention between less relevant pairs based on the distance, providing a simplified approach to modeling complex molecular structures as follows (the case of $graph$ block): 


(3a) \begin{align*} Q_{i} = x^{\mathit{graph}^{l-1}} W_{i}^{Q}, & \ K_{i} = x^{\mathit{graph}^{l-1}} W_{i}^{K}, \ V_{i} = x^{\mathit{graph}^{l-1}} W_{i}^{V} \end{align*}

(3b) \begin{align*} head_{i} & = \mathrm{Softmax}\left(\frac{Q_{i} K_{i}^{T}}{\sqrt{d_{\mathit{model}}}}\right) V_{i}, \end{align*}

(3c) \begin{align*} multihead & = \mathrm{Concat}(head_{1},..., head_{h}) W^{O}, \end{align*}

(3d) \begin{align*} focus & = multihead \odot Strength^{\mathit{graph}}, \end{align*}

(3e) \begin{align*} residual & = \mathrm{LayerNorm}(x^{\mathit{graph}^{l-1}} + focus), \end{align*}

(3f) \begin{align*} x^{\mathit{graph}^{l}} & = \mathrm{LayerNorm}(residual + \mathrm{FFN}(residual)), \end{align*}

where $x^{\mathit{graph}^{l-1}}$ and $x^{\mathit{graph}^{l}}$ are the updated latent features of the $graph$ block in ($l-1$)-th and $l$-th layers, respectively, $h$ is the head number of multi-head attention, and $W_{i}^{Q} \in \mathbb{R}^{d_{\mathit{model}} \times \mathit{d_{\mathit{model}}/h}}$, $W_{i}^{K} \in \mathbb{R}^{d_{\mathit{model}} \times \mathit{d_{\mathit{model}}/h}}$, $W_{i}^{V} \in \mathbb{R}^{d_{\mathit{model}} \times \mathit{d_{\mathit{model}}/h}}$, and $W^{O} \in \mathbb{R}^{d_{\mathit{model}} \times d_{\mathit{model}}}$ are trainable parameters. In the case of the $conf$ block, $x^{\mathit{conf}^{l}}$ can calculated from $x^{\mathit{conf}^{l-1}}$ and $Strength^{\mathit{conf}}$ in the same process as the $graph$ block.

Finally, the outputs $x^{\mathit{graph}^{\mathit{out}}}$ and $x^{\mathit{conf}^{\mathit{out}}}$ of the two blocks were weighted using the hyperparameter $\lambda _{g}$ and the concatenated feature vector was used to derive the final output $out^{\mathit{atom}}$ of the atom model as follows: 


(4) \begin{align*}& out^{\mathit{atom}} = \mathrm{Linear}(\mathrm{Concat}(\lambda_{g} * x^{\mathit{graph}^{\mathit{out}}}, (1 - \lambda_{g}) * x^{\mathit{conf}^{\mathit{out}}})).\end{align*}

### Monomer model

We constructed a monomer model based on the $16$ types of monomer descriptors to capture the partial structural information of peptides at the sequence level (Fig. 3 (B)). The CNN was used to learn partial structural features and sequence information. For the convolution layer, we used the general 1D-CNN layer or a CyclicConv layer [38] that considers peptide circularity. The use of the CyclicConv or 1D-CNN layer was determined by hyperparameter tuning. Finally, the monomer model derived the latent feature $out^{\mathit{monomer}}$.

### Peptide model

To capture the characteristics of the entire molecule, we used $16$ peptide descriptors representing physicochemical properties and $2048$-bit Morgan FP ($1024$-bit, radius: $2$; $1024$-bit, radius: $3$) representing substructural information as peptide-level features. The descriptor and Morgan FP were each trained with different multilayer perceptrons (MLPs) and the latent feature vectors $x^{\mathit{desc}^{\mathit{out}}}$ and $x^{\mathit{fp}^{\mathit{out}}}$ were concatenated and used to derive the final output $out^{\mathit{peptide}}$ of the peptide model as follows: 


(5) \begin{align*}& out^{\mathit{peptide}} = \mathrm{Linear}(\mathrm{Concat}(x^{\mathit{desc}^{\mathit{out}}}, x^{\mathit{fp}^{\mathit{out}}})).\end{align*}

### Fusion model

As shown in Fig. 2, the output latent feature vectors $out^{\mathit{atom}}$, $out^{\mathit{monomer}}$, and $out^{\mathit{peptide}}$ of the three sub-models were concatenated to generate the final molecular feature vector, which was passed through a shared layer for the final permeability prediction $out^{\mathit{fusion}}$. As the model becomes more complex, problems such as gradient disappearance may occur, causing input information to not be transmitted. Auxiliary loss is a learning technique in which additional losses are incurred to optimize the NN learning process. Directly propagating errors to the middle network layer can prevent gradient disappearance and improve embedding and learning efficiency [61]. Hence, we designed the three sub-model losses $L_{\mathit{atom}}$, $L_{\mathit{monomer}}$, and $L_{\mathit{peptide}}$ derived from the output of each sub-model (the definition of $L_{\mathit{monomer}}$ is shown in Equation (6a)), and layer losses $L_{\mathit{layer}_{a}}$, $L_{\mathit{layer}_{m}}$, and $L_{\mathit{layer}_{p}}$ derived from the averaged outputs of the layers in each block (Transformer, CNN, and MLP) of the three sub-models (the definition of $L_{\mathit{layer}_{m}}$ is shown in Equation (6b)) in addition to the main loss $L_{\mathit{fusion}}$ calculated from the output of the fusion model: 


(6a) \begin{align*} L_{\mathit{monomer}} & = \mathrm{Lossfunc}(\mathrm{Linear}(out^{\mathit{monomer}})), \end{align*}

(6b) \begin{align*} L_{\mathit{layer}_{m}} & = \mathrm{Lossfunc}(\mathrm{Linear}(\mathrm{Mean}(x^{\mathit{mono^{1}}},..., x^{\mathit{mono^{num-cnn}}}))). \end{align*}

The loss function during training is presented in Equation (7): 


(7) \begin{align*}& \begin{split} Loss = L_{\mathit{fusion}} &+ \gamma_{\mathit{sub}} * (L_{\mathit{atom}} + L_{\mathit{monomer}} + L_{\mathit{peptide}}) \\ &+ \gamma_{\mathit{layer}} * (L_{\mathit{layer}_{a}} + L_{\mathit{layer}_{m}} + L_{\mathit{layer}_{p}}), \end{split}\end{align*}

where the weight parameter $\gamma _{\mathit{sub}}$ was set to $0.10$ and $\gamma _{\mathit{layer}}$ was set to $0.05$. Only the output value $out^{\mathit{fusion}}$ of the fusion model was used during inference. The hyperparameters of CycPeptMP were determined by $150$ trials using Optuna software [62] based on the average RMSE of three runs; the search range and results are shown in Supplementary Table S1, and hyperparameters with a significant impact are shown in Supplementary Fig. S3.

### Data augmentation

**Figure 4:**
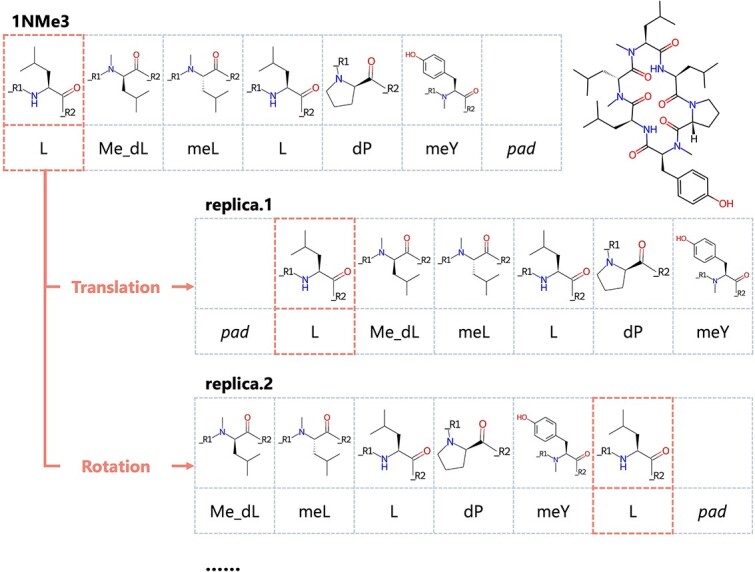
Sequence arrangement in the monomer model. The aligned monomer descriptors were translated and rotated based on the sequence information.

Although the amount of available biological data has increased, experimental data remains limited compared to data for natural language processing and computer vision. For example, the Tox21 dataset deals with the toxicity classification of small molecules and has only approximately $8000$ data points. This limitation in biological data, particularly the scarcity of data with measurement values, has motivated the increased use of self-supervised learning approaches, such as contrastive learning [63] and pre-training [44, 45]. These methods are commonly employed in scenarios where labeled data is scarce while large amounts of unlabeled structural data are available. However, these techniques remain challenging for cyclic peptides given a more limited availability compared to small molecules. Apart from these techniques, data augmentation (such as oversampling and data warping) has been commonly used in the image processing field to increase training efficiency when the data are insufficient. The augmented data represent a more comprehensive set of possible data points that minimizes the distance between the training and any future testing sets and reduces the risk of overfitting [64]. We used three augmentation methods to generate $60$ replicas based on the properties of SMILES, the nature of cyclic peptide sequences, and the complexity of cyclic peptide conformational changes to improve the learning efficiency of the model. First, the SMILES enumeration technique [65] was used to permute the atom order and generate input for the atom model with a different ordering. Subsequently, the input of the monomer model was rearranged using sequence arrangement considering the circularity of the cyclic peptide—the aligned monomer descriptors were augmented by the combination of sequence translation and rotation (change the start point of sequence) as shown in Fig. 4. Finally, considering the complex conformational changes during membrane permeation of cyclic peptides, $60$ different conformations per peptide/monomer were generated using RDKit to incorporate more diverse 3D information into the model. Cyclic peptide conformations were used to calculate the $Conf$ matrix for the atom model and peptide descriptors for the peptide model. Monomer conformations were used to calculate the monomer descriptors for the monomer model. Introducing variations of the input data enables our model to become more robust to cyclic peptide conformational flexibility and allows it to partially consider circularity. Data augmentation effectively increases the size of the training set, leading to more efficient and stable training. During training, each replica was given the same label and treated as independent data. During inference, relying on a single conformation could introduce bias, considering the conformational flexibility of cyclic peptides. Therefore, the average of $60$ replicas was used as the final predicted value to represent the conformational ensemble of peptides.

### Baseline methods

We validated the performance of CycPeptMP based on comparisons with seven baseline methods.

- Three traditional baselines: We constructed an RF model with $2048$-bit Morgan FP, a support vector machine (SVM) model with seven 2D peptide descriptors, and an SVM model with $16$ 2D and 3D peptide descriptors to represent traditional cyclic peptide membrane permeability prediction methods. The hyperparameters of the RF and SVM models were determined by a grid search (Supplementary Table S2).
- Two transformer-based methods: MAT [50] and SAT [47] were compared as state-of-the-art transformer-based methods for predicting small-molecule properties. MAT augments the transformer’s self-attention mechanism with domain-specific knowledge, incorporating inter-atomic distances and molecular graph structure into the attention calculation to capture structural information. SAT focuses on the problem of traditional transformers in that positional encoding does not necessarily capture the structural similarity between nodes. It proposes a structure-aware transformer that incorporates structural information into self-attention by extracting a subgraph representation rooted at each node before computing the attention.
- Two multi-level feature methods: PharmHGT [49] designs features on the atom and fragment levels and constructs a heterogeneous graph considering the correspondence between atoms and fragments for a transformer-based model. FinGAT [41] uses a GAT model to extract atom-level information and combines it with Morgan FP to capture the molecular structure from multiple perspectives.

The hyperparameters of four DL-based models were determined by $50$ trials using Optuna software based on the average RMSE of three runs (Supplementary Table S3).

**Table 3:** Performance comparison between seven baseline methods and CycPeptMP using the test set. The metrics are the averaged values of three repeated runs; the best result for each metric is indicated in bold

Metrics	RF	SVM-2D	SVM-2D3D	MAT	SAT	PharmHGT	FinGAT	CycPeptMP
MAE	$0.485 \pm 0.003$	$0.488 \pm 0.005$	$0.418 \pm 0.001$	$0.538 \pm 0.064$	$0.690 \pm 0.053$	$0.485 \pm 0.033$	$0.493 \pm 0.008$	$\mathbf{0.355 \pm 0.007}$
MSE	$0.380 \pm 0.004$	$0.449 \pm 0.014$	$0.345 \pm 0.002$	$0.503 \pm 0.102$	$0.879 \pm 0.155$	$0.420 \pm 0.032$	$0.415 \pm 0.021$	$\mathbf{0.253 \pm 0.013}$
R	$0.815 \pm 0.003$	$0.781 \pm 0.007$	$0.834 \pm 0.001$	$0.753 \pm 0.044$	$0.569 \pm 0.068$	$0.800 \pm 0.019$	$0.800 \pm 0.007$	$\mathbf{0.883 \pm 0.003}$
$\mathrm{R^{2}}$	$0.657 \pm 0.003$	$0.595 \pm 0.012$	$0.689 \pm 0.002$	$0.547 \pm 0.091$	$0.207 \pm 0.140$	$0.622 \pm 0.029$	$0.626 \pm 0.018$	$\mathbf{0.772 \pm 0.011}$

## Results and Discussion

### Performance comparison for the test set

**Figure 5:**
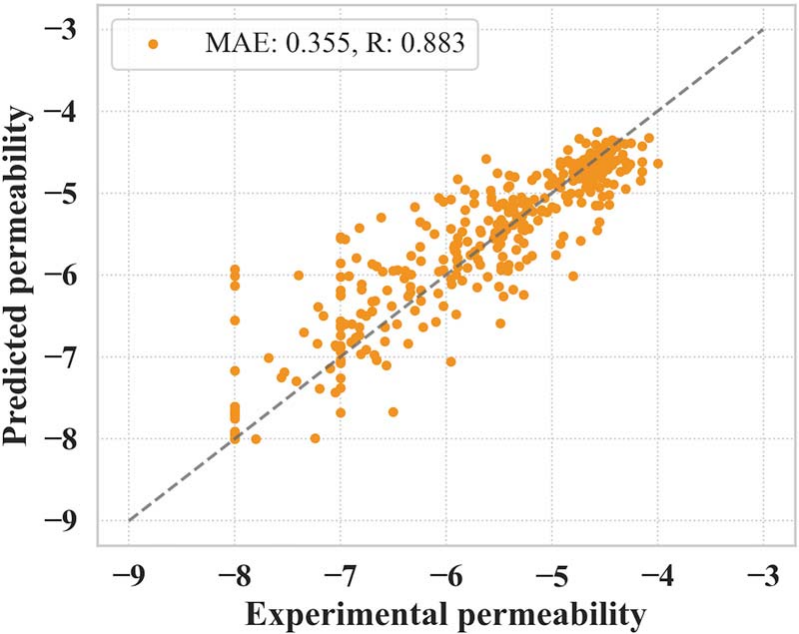
CycPeptMP prediction results for the test set. The predicted value of the test set is the average value of three runs.

The prediction accuracy results for the test set are shown in Table 3 (the prediction accuracy and results for the validation set are shown for reference purposes in Supplementary Table S4 and Supplementary Fig. S4). CycPeptMP ranked first in all evaluation metrics, reflecting a significant improvement in prediction performance over all existing methods ($\mathrm{MAE}=0.355$, Fig. 5). Considering the structural diversity of the test set, CycPeptMP showed good generalization performance and could learn the complex structures of cyclic peptides, which is difficult to apply pre-training through augmentation. The RF model constructed based on Morgan FP showed good prediction performance and ranked third among all methods ($\mathrm{MAE}=0.485$, Supplementary Fig. S5 (A)). SVM with 2D peptide descriptors ($\mathrm{MAE}=0.488$, Supplementary Fig. S5 (B)) had lower prediction accuracy than the RF model, whereas SVM with 3D descriptors improved prediction accuracy, making it superior to the RF model for the test set ($\mathrm{MAE}=0.418$, Supplementary Fig. S5 (C)). Cyclic peptide membrane permeation by passive diffusion negatively correlated with molecule size. SVM could partially predict permeability by using lipophilicity descriptors, such as LogP, which are largely dependent on molecular weight. CycPeptMP effectively combined Morgan FP and $16$ 2D and 3D peptide descriptors as peptide-level information to comprehensively characterize peptide structures from a topological and physicochemical perspective, leading to an improvement in prediction capabilities. Neither graph representation transformer-based MAT ($\mathrm{MAE}=0.538$, Supplementary Fig. S5 (D)) nor SAT ($\mathrm{MAE}=0.690$, Supplementary Fig. S5 (E)) could predict membrane permeability. Although MAT and SAT are state-of-the-art methods for predicting small-molecule properties, they could not effectively learn the structures of more complex cyclic peptides since they only utilize the atom-level information without augmentation technique. In addition to atom-level information, PharmHGT with fragment MACCS Keys ($\mathrm{MAE}=0.485$, Supplementary Fig. S5 (F)) and FinGAT with molecular Morgan FP ($\mathrm{MAE}=0.493$, Supplementary Fig. S5 (G)) had significantly improved prediction accuracies compared to MAT and SAT, with the same level of accuracy as the RF model and 2D SVM. Hence, designing features from various perspectives may be key to successfully predicting the membrane permeability of cyclic peptides. These findings indicated that CycPeptMP effectively employed three levels of features to capture a wide range of structural information from the smallest atomic detail to the broader peptide-level conformation.

Meanwhile, different experimental conditions can significantly alter the measurements. CycPeptMPDB records all reported values from different literature assays for the same peptide (this study used values from the most recent literature). For example, cyclosporin A is a peptide with PAMPA measurements reported from five literature sources with permeabilities of $-5.01$, $-6.2$, $-6.15$, $-5.71$, and $-5.72$ (max: $-5.01$, min: $-6.2$, std: $0.427$) in chronological order of publication; 1NMe3 is a peptide with PAMPA measurements reported from six literature sources with permeabilities of $-4.5$, $-4.4$, $-6$, $-6.24$, $-6.4$, and $-5.52$ (max: $-4.4$, min: $-6.4$, std: $0.798$) in chronological order of publication. Since these errors are already present in the measurement experiment, the prediction accuracy $\mathrm{MAE}=0.355$ of CycPeptMP may be close to the limit of prediction. The predicting results of other assays using the model trained with PAMPA are shown in Supplementary Table S5.

### Lower limit processing of permeability

We rounded the permeability with $-10\leq \mathrm{LogP_{exp}}<-8$ to $-8$ because the detection limit for most literature is $-8$. Among them, most peptides were recorded in CycPeptMPDB with $\mathrm{LogP_{exp}}=-10$. Most were not measured as $-10$ and were set to $-10$ by CycPeptMPDB as there was no clear value. Therefore, their membrane permeability was unreliable. To discuss the effect of these data, we calculated the accuracy of $12$ peptides with $\mathrm{LogP_{exp}}=-10$, $6$ peptides with $-10<\mathrm{LogP_{exp}}\leq -8$, and $326$ other peptides with $-8<\mathrm{LogP_{exp}}$ of the test set, respectively. Peptides with $\mathrm{LogP_{exp}}=-10$ could not be predicted by any method ($\mathrm{MAE}=0.766$ to $1.239$, Supplementary Fig. S5). We have included these unreliable experimental values in our data to incorporate as much data as possible; however, it may be more appropriate to eliminate them. For the peptides that have clear measurement values with $-10<\mathrm{LogP_{exp}}\leq -8$, no baseline methods except FinGAT ($\mathrm{MAE}=0.353$) could predict them ($\mathrm{MAE}=0.564$ to $1.022$). Nevertheless, CycPeptMP accurately predicted them ($\mathrm{MAE}=0.265$), further demonstrating the superiority of the proposed method over baselines.

### Performance comparison of $10$-fold cross-validation

Considering generalization performance, the Kennard–Stone algorithm was employed to maximize the distance between data points in the chemical space of the test set to extract the most diverse test set possible from the CycPeptMPDB data. For multiple random sampling evaluation, we performed a new $10$-fold cross-validation with different random seeds for each run without altering the determined hyperparameters. As shown in Table 4, CycPeptMP consistently demonstrated the highest prediction performance for the difficult-to-predict test set ($\mathrm{MAE}=0.355$) and $10$-fold cross-validation ($\mathrm{MAE}=0.352$). All baseline methods had higher accuracy for the $10$-fold cross-validation than the test set (the prediction performance of each model was relatively the same as the original validation set shown in Supplementary Table S4).

**Table 4:** Performance comparison between seven baseline methods and CycPeptMP by $10$-fold cross-validation. The metrics are the averaged values of ten repeated runs; the best result for each metric is indicated in bold

Metrics	RF	SVM-2D	SVM-2D3D	MAT	SAT	PharmHGT	FinGAT	CycPeptMP
MAE	$0.400 \pm 0.010$	$0.396 \pm 0.010$	$0.386 \pm 0.008$	$0.453 \pm 0.040$	$0.459 \pm 0.061$	$0.399 \pm 0.024$	$0.408 \pm 0.022$	$\mathbf{0.352 \pm 0.015}$
MSE	$0.318 \pm 0.021$	$0.349 \pm 0.022$	$0.334 \pm 0.020$	$0.394 \pm 0.047$	$0.413 \pm 0.070$	$0.316 \pm 0.032$	$0.328 \pm 0.022$	$\mathbf{0.271 \pm 0.023}$
R	$0.748 \pm 0.018$	$0.725 \pm 0.017$	$0.739 \pm 0.015$	$0.690 \pm 0.034$	$0.689 \pm 0.058$	$0.758 \pm 0.023$	$0.751 \pm 0.020$	$\mathbf{0.786 \pm 0.019}$
$\mathrm{R^{2}}$	$0.557 \pm 0.028$	$0.516 \pm 0.027$	$0.537 \pm 0.025$	$0.453 \pm 0.061$	$0.427 \pm 0.094$	$0.560 \pm 0.043$	$0.545 \pm 0.028$	$\mathbf{0.613 \pm 0.033}$

### Ablation study of atom and monomer models

**Figure 6:**
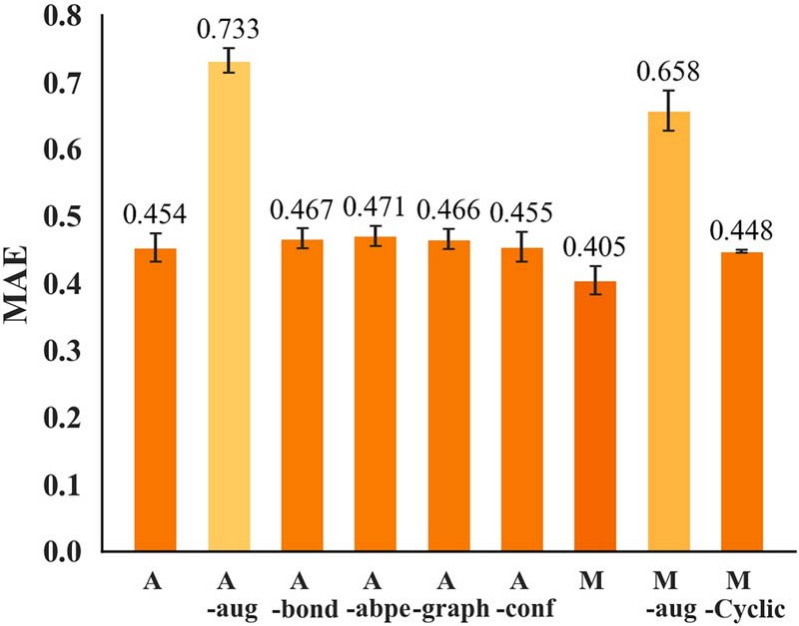
Ablation results (MAE) for the atom and monomer models using the test set.

We conducted ablation studies on atom and monomer models with complex architectures (Fig. 6; the validation results are shown in Supplementary Fig. S6 (A)). For the atom model, A is the original model, and A–aug is the result without data augmentation. We measured the prediction accuracy when not using the $Bond$ matrix (A–bond), using ordinary absolute positional encoding [66] instead of the $Bond$ matrix (A–abpe), retaining only the $Conf$ block (A–graph), or retaining only the graph block (A–conf). As shown in Fig. 6, the prediction accuracy of the atom model significantly improved by augmentation (A: $0.454$, A–aug: $0.733$). Regarding the architectural changes of the atom model, the original A showed the highest prediction accuracy, while the deletion of any element decreased the prediction accuracy. The relationship between atoms was captured more effectively using $Bond$ (A: $0.454$) than absolute positional encoding (A–abpe: $0.471$), and the impact of removing $Graph$ block (A–graph: $0.466$) was greater than that of removing $Conf$ block (A–conf: $0.455$).

For the monomer model, M is the original model and M–aug is the result without data augmentation. We also measured the accuracy change when replacing the general 1D-CNN layers with CyclicConv layers (M–Cyclic). Similar to the atom model results, the prediction accuracy of the monomer model significantly improved by augmentation (M: $0.405$, M–aug: $0.658$). These results showed that SMILES enumeration for the atom model and sequence arrangement for the monomer model effectively improved learning efficiency. Moreover, the augmentation technique is essential for learning the complex structure of cyclic peptides. Additionally, the 1D-CNN layer (M: $0.405$) was superior to the CyclicConv layer (M–Cyclic: $0.448$), consistent with previous findings [38].

### Ablation study of the fusion model

**Figure 7:**
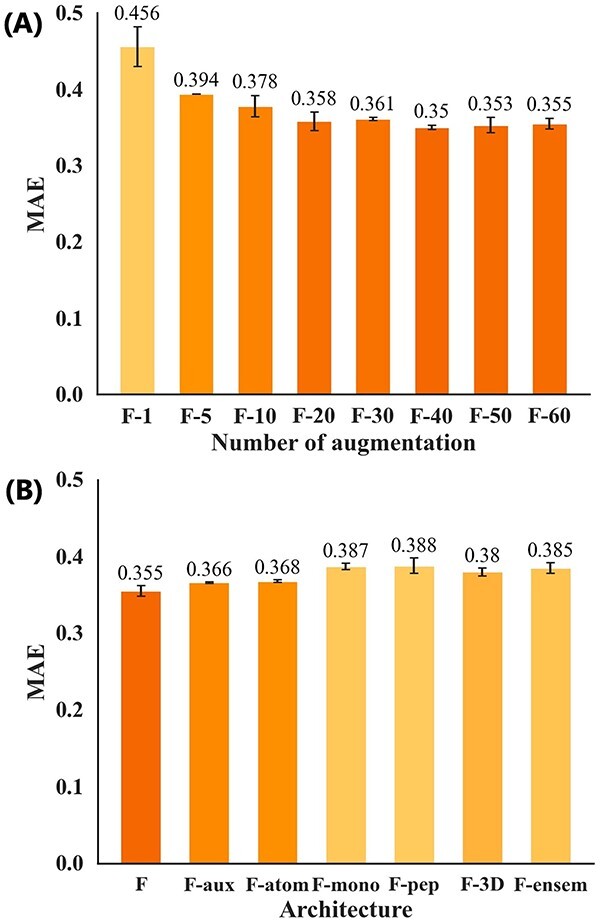
Ablation results (MAE) for the fusion model using the test set. (A) Different numbers of input replicas. (B) Different architectures.

The ablation study for the fusion model measured the influences of the number of replicas generated by augmentation and changes in architecture. Figure 7 (A) shows the accuracy based on $1$ (no augmentation), $5$, $10$, $20$, $30$, $40$, $50$, and $60$ (CycPeptMP) replicas per peptide. We observed a significant improvement in prediction accuracy compared to that without augmentation (F–1: $0.456$) even with five replicas (F–5: $0.394$). However, over $20$ replicas showed approximately the same prediction accuracy as the amount of training data increased. This may be due to the limitations of increased diversity caused by merely reordering the inputs and the lack of diversity in the generated conformations (prediction results of the test set using regenerated conformations are shown in Supplementary Table S6).

In Fig. 7 (B), F is the original CycPeptMP model; F–aux is the model without auxiliary loss; F–atom, F–mono, and F–pep represent the models lacking the respective sub-models; F–3D is the model that did not use all 3D information ($Conf$ and 3D descriptors); and F–ensem represents the model with each sub-model allowed to directly predict membrane permeability and the average ensemble of three predictions was taken. Auxiliary loss improved prediction accuracy (F–aux: $0.366$). Furthermore, prediction accuracy decreased when any of the three sub-models were removed, indicating that the three levels of information were important to predicting membrane permeability. The peptide model had the greatest influence (F–pep: $0.388$), followed by the monomer (F–mono: $0.387$) and atom model (F–atom: $0.368$). The use of 3D information insignificantly improved prediction accuracy (F–3D: $0.38$). Accuracy may be improved by generating conformations using a more rigorous method, such as MD simulations. Correctly addressing the possible conformational distribution of peptides appears important. A detailed discussion of closed-conformation for the RDKit-generated conformations is presented in Supplementary Fig. S7. Finally, the average prediction accuracy further decreased when using a sub-model ensemble (F–ensem: $0.385$). Hence, it was better to extract latent features than having each sub-model directly predict permeability.

### Comparison with MD-based method

Cyclic peptides tend to exist in various conformations, resulting in slow conformational transitions relative to simulation time scales. The first MD-based large-scale prediction of cyclic peptide membrane permeability used steered MD and replica-exchange umbrella sampling to accelerate sampling and simulated the membrane permeation process of $100$ six-residue and $56$ eight-residue peptides through a lipid bilayer [30]. We compared their prediction results with the CycPeptMP results for $23$ peptides (Supplementary Table S7) included in three validation sets ($16$ peptides) and the test set ($7$ peptides).

**Figure 8:**
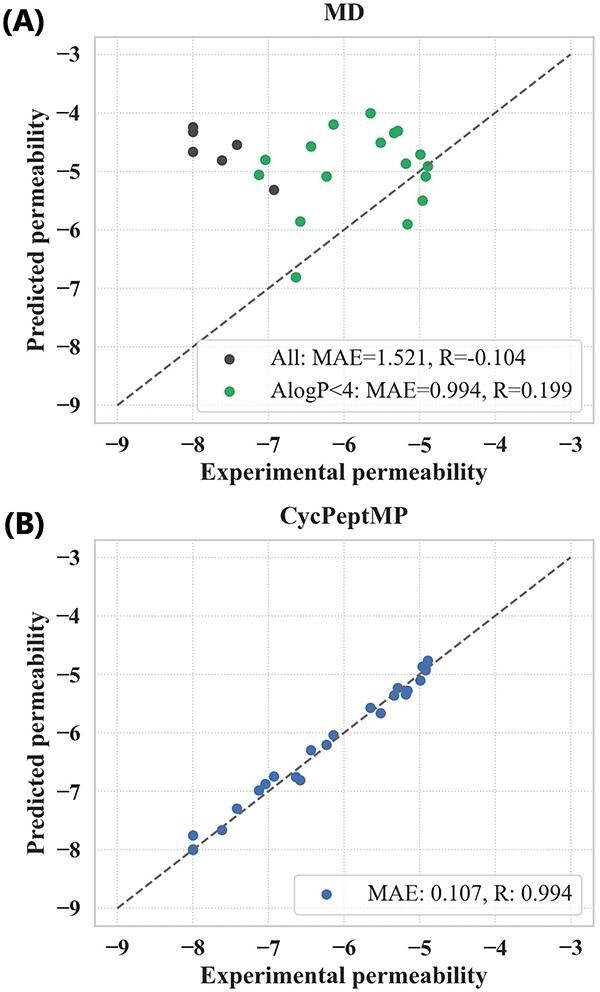
(A) Prediction results of the MD-based method. Black dots represent hydrophobic peptides with $\mathrm{AlogP} \geq 4$; green dots represent the remaining peptides with $\mathrm{AlogP}<4$. (B) Prediction results of CycPeptMP.

While the MD-based method could not successfully predict the membrane permeability of these $23$ peptides ($\mathrm{MAE}=1.521$), CycPeptMP accurately predicted them all ($\mathrm{MAE}=0.107$) (Fig. 8). Hydrophobic cyclic peptides have insufficient solubility, slowly diffuse in the unstirred water layer, and are likely adsorbed to the membrane. Therefore, these behaviors could not be reproduced using the inhomogeneous solubility-diffusion model (ISDM), which only considers direct membrane permeation processes [30]. They reported a prediction accuracy ($\mathrm{R}$) of only $0.21$ for all $100$ six-residue peptides; however, the accuracy increased to $0.54$ when $33$ hydrophobic peptides ($\mathrm{AlogP} \geq 4$) were excluded. A similar trend was observed among the $23$ peptides compared in this study: the $\mathrm{MAE}$ of the MD-based method improved from $1.521$ to $0.994$ by excluding six peptides with $\mathrm{AlogP} \geq 4$. Overall, CycPeptMP can accurately and rapidly predict peptide permeability with superior performance compared to MD-based methods, representing a promising tool for cyclic peptide drug discovery.

## Conclusion

CycPeptMP represents a high-performance deep learning-based technique for predicting the membrane permeability of cyclic peptides. It incorporates atom-, monomer-, and peptide-level features and improves training efficiency through three types of data augmentation techniques. CycPeptMP exhibits excellent prediction accuracy and generalization performance compared to existing methods. Moreover, we confirmed that CycPeptMP accurately predicts the permeability of peptides with much lower computational costs where MD-based methods fail. With its ability to rapidly identify high-permeability peptides, CycPeptMP has the potential to significantly advance cyclic peptide drug discovery. It also paves the way for the development of more effective DL-based techniques in related fields. Future studies should focus on improving the prediction performance by generating 3D conformations with a more rigorous method.

**Key Points**

- This study presents CycPeptMP, a novel DL-based method to predict the membrane permeability of cyclic peptides. CycPeptMP utilizes a multi-level feature design and data augmentation to simplify the characterization of complex peptide structures and improve model performance.
- CycPeptMP achieves excellent performance using a test set containing various structures, demonstrating the functionality gained by implementing the three-level feature-appropriate architectural design.
- CycPeptMP effectively determines the permeability of peptides that is difficult to predict using MD-based methods and can promote the efficacy of cyclic peptide drug discovery in myriad research directions, such as structure–activity relationship analysis and lead optimization.

## Supplementary Material

Supporting_Information_bbae417

## Data Availability

The structures and membrane permeability data of the cyclic peptides used in this study were sourced from CycPeptMPDB [51] (http://cycpeptmpdb.com/). The code has been published on GitHub (https://github.com/akiyamalab/cycpeptmp).
