# Supplementary material for: CycPeptMP: enhancing membrane permeability prediction of cyclic peptides with multi-level molecular features and data augmentation
Source: Brief Bioinform. 2024 Aug 29;25(5):bbae417. doi: 10.1093/bib/bbae417 (PMC11361855; doi:10.1093/bib/bbae417)
Supplement: Supporting_Information_bbae417 [file supporting_information_bbae417.pdf]

Supporting Information

CycPeptMP: Enhancing Membrane Permeability Prediction of Cyclic Peptides with Multi-Level Molecular Features and Data Augmentation

Jianan Li[1], Keisuke Yanagisawa[1, 2], and Yutaka Akiyama[1, 2, *]

[1]Department of Computer Science, School of Computing,
Tokyo Institute of Technology, Meguro-ku, Tokyo 152-8550, Japan
[2]Middle-Molecule IT-based Drug Discovery Laboratory (MIDL),
Tokyo Institute of Technology, Meguro-ku, Tokyo 152-8550, Japan

* To whom correspondence should be addressed.
Email: akiyama@c.titech.ac.jp

1 Supplemental Figures

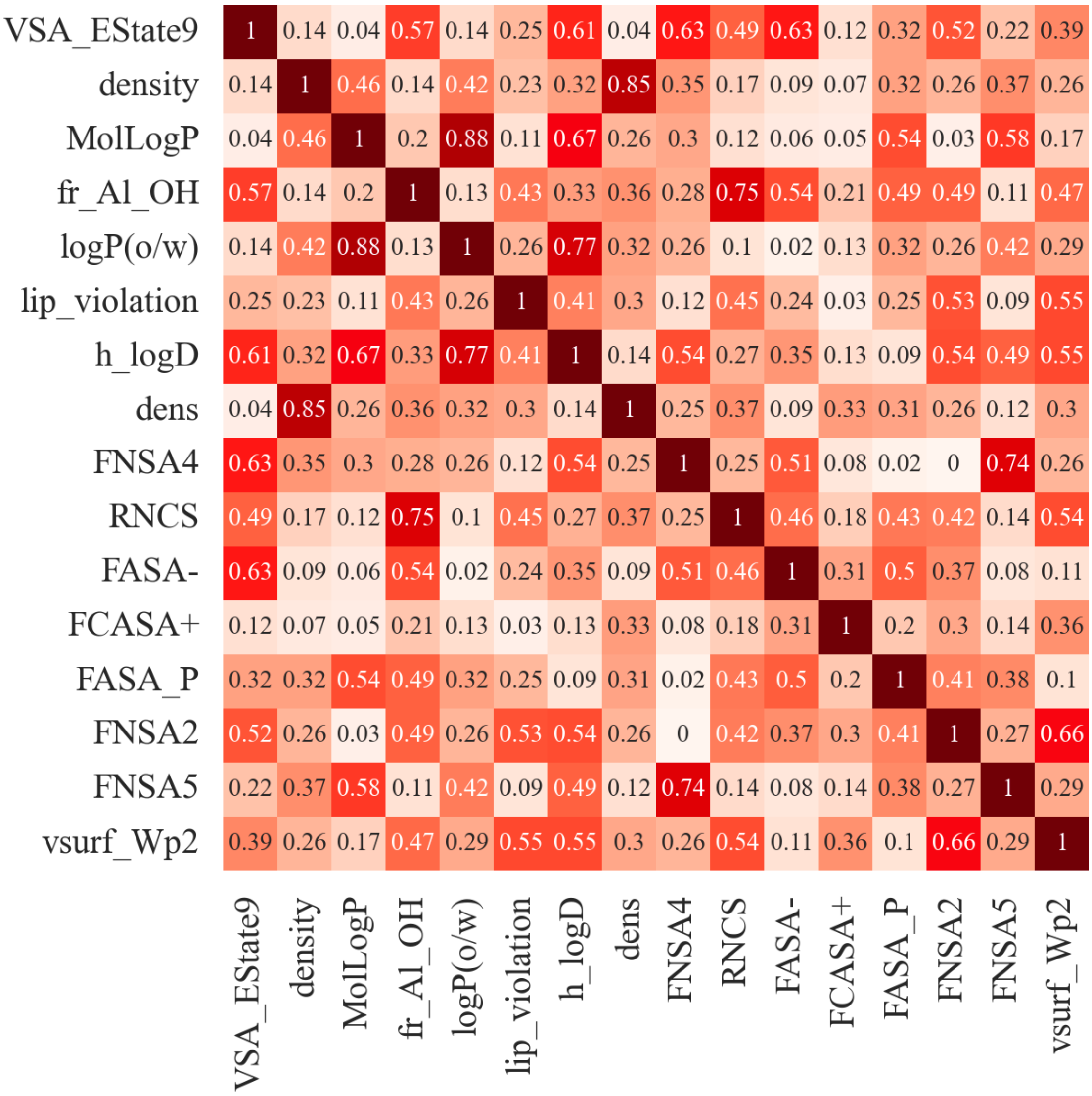

Figure S1: Heat map of absolute correlation coefficient values for 16 selected peptide descriptors. The pair with the highest correlation is MolLogP and logP(o/w) (|R| = 0.884).

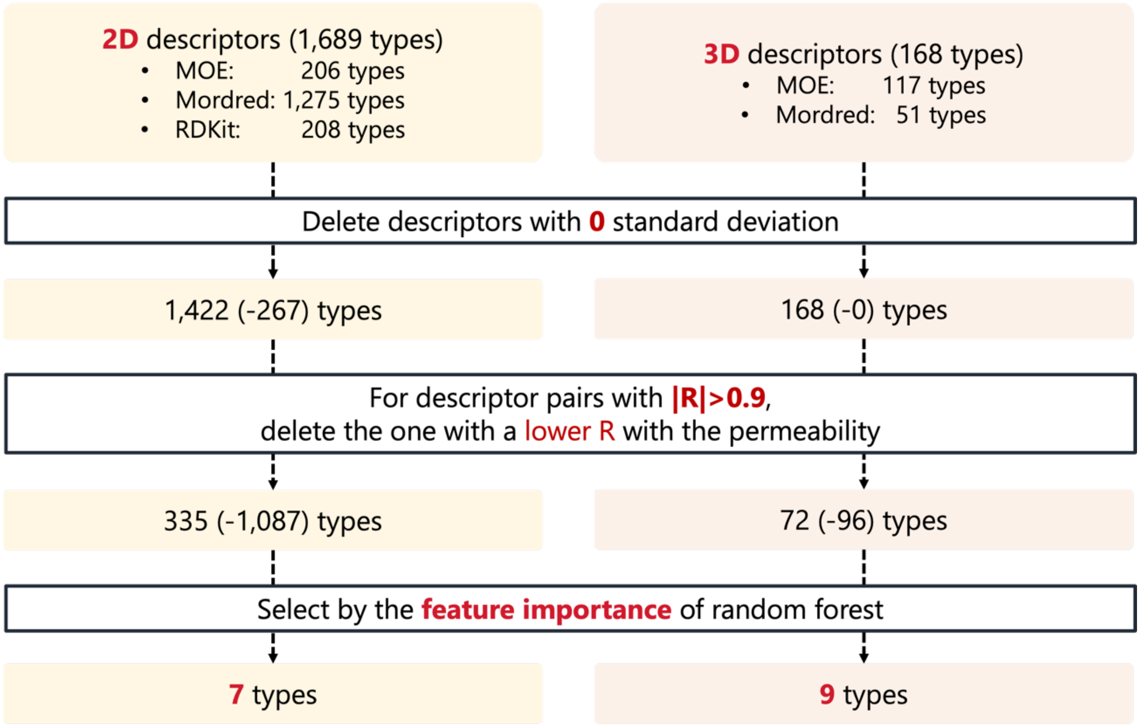

Figure S2: Preprocessing and selection of peptide descriptors. 206 2D descriptors and 117 3D descriptors were calculated by MOE [1], 1275 2D and 51 3D descriptors that could be calculated correctly were calculated by Mordred [2], and 208 2D descriptors were calculated by RDKit [3]. Finally, seven 2D peptide and nine 3D peptide descriptors were selected based on the feature importance of two random forest models.

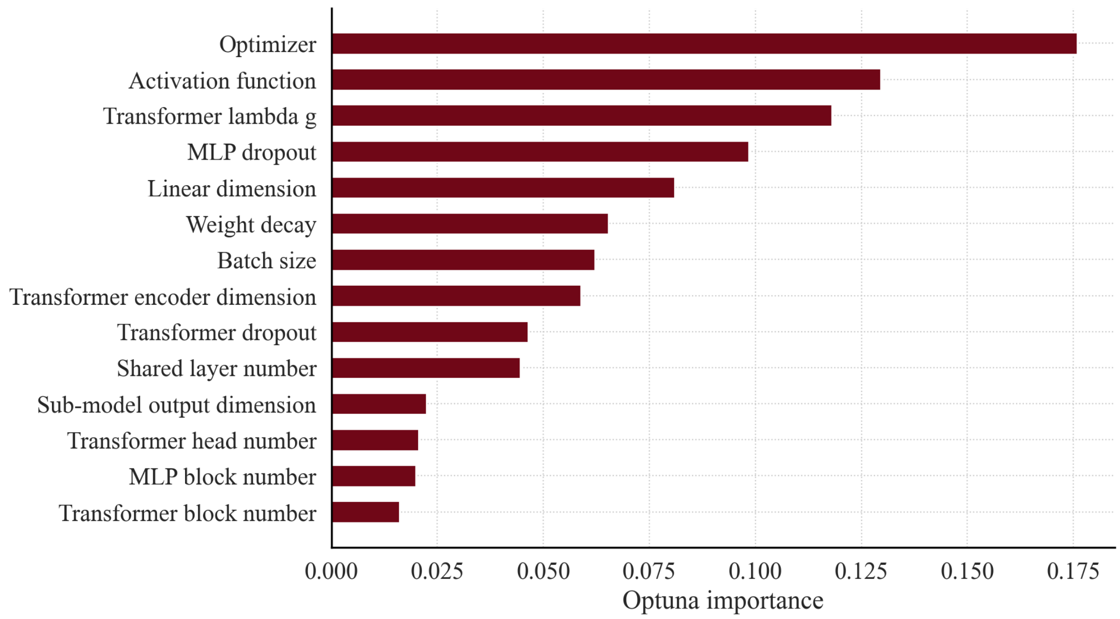

Figure S3: Top 14 hyperparameters with an Optuna [4] importance > 0.01 on the CycPeptMP hyperparameter search. Optuna importance is calculated based on the fANOVA hyperparameter importance evaluation algorithm [5]; the sum of the importance values is normalized to 1.0.

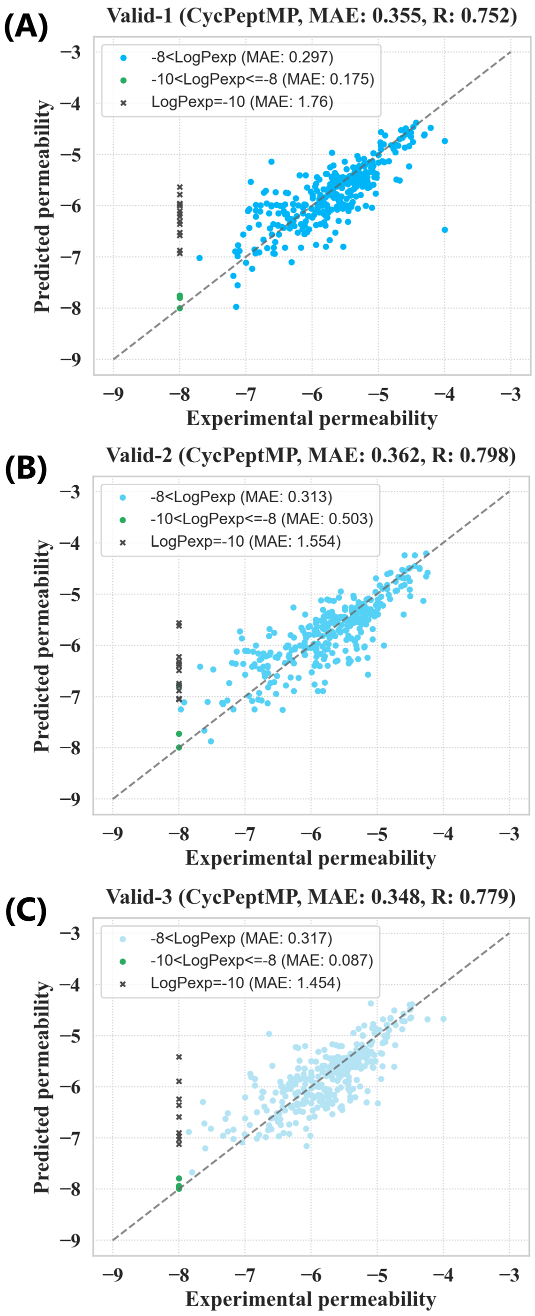

Figure S4: CycPeptMP prediction results of the (A) 1st validation set (Valid-1), (B) 2nd validation set (Valid-2), and (C) 3rd validation set (Valid-3). Each figure shows the MAE and R of all data and the MAE calculated from peptides with $LogP_{exp}$ recorded in CycPeptMPDB of -10 (could not be correctly measured), peptides with $-10 < LogP_{exp} \leq -8$ (correctly measured and rounded to -8, the lower limit), and other peptides with $-8 < LogP_{exp}$. Similar to the test set results, peptides with $LogP_{exp} = -10$ could not be predicted, and peptides with $-10 < LogP_{exp} \leq -8$ showed the same level of accuracy as others.

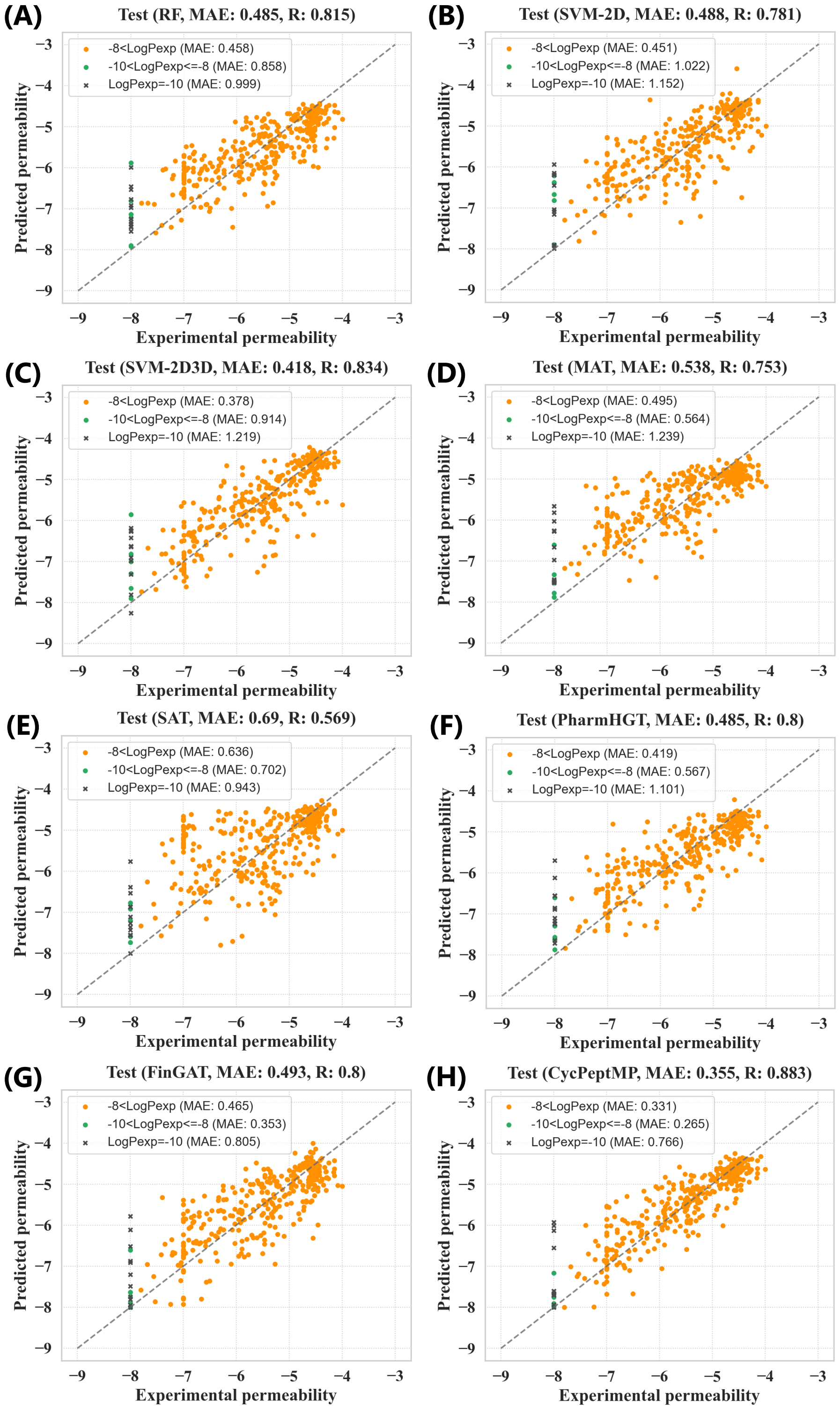

Figure S5: Prediction results of the test set by seven baseline methods and CycPeptMP. (A) RF model, (B) SVM-2D model, (C) SVM-2D3D model, (D) MAT model, (E) SAT model, (F) PharmHGT model, (G) FinGAT model, and (H) CycPeptMP. The predicted value of the test set is the average value of three runs.

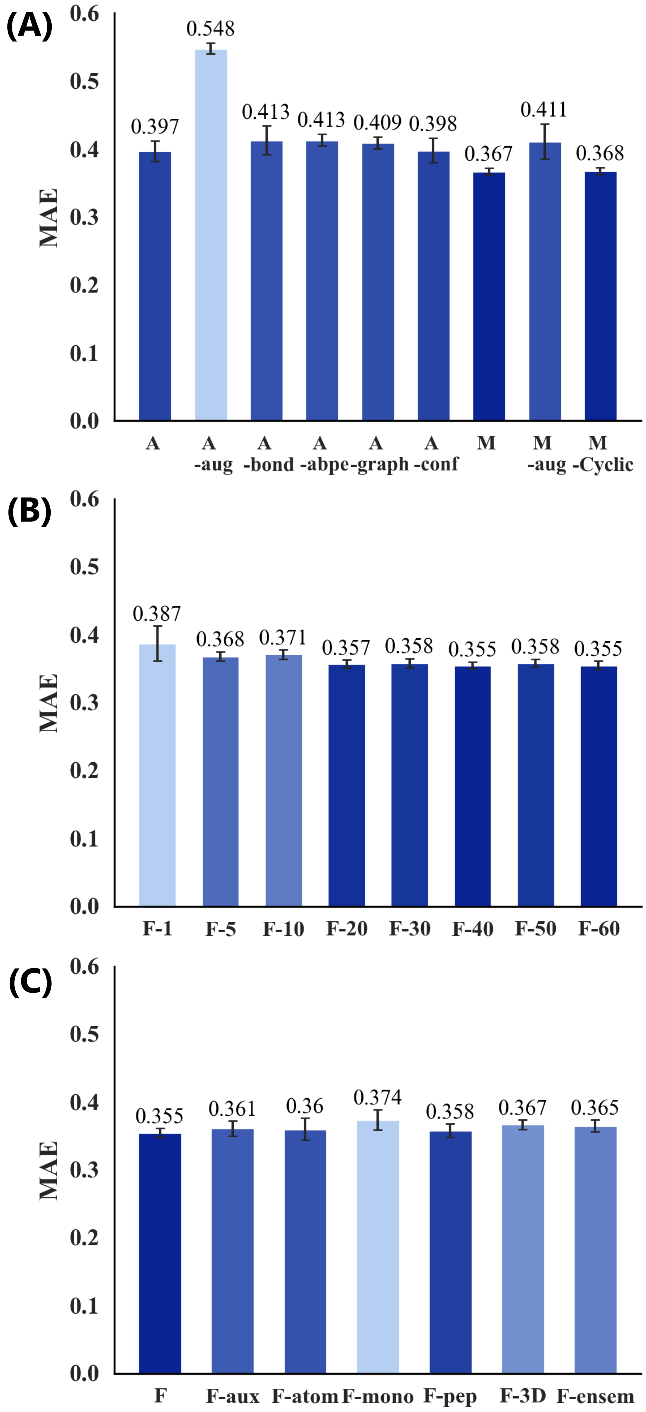

Figure S6: Ablation results (MAE) for the atom and monomer models using the validation set (A) and ablation results for the fusion model (B, C). (A) Effects of changing the number of input replicas and architecture for the atom and monomer models. (B) Effects of changing the number of input replicas for the fusion model. (C) Effects of changing the architecture for the fusion model.

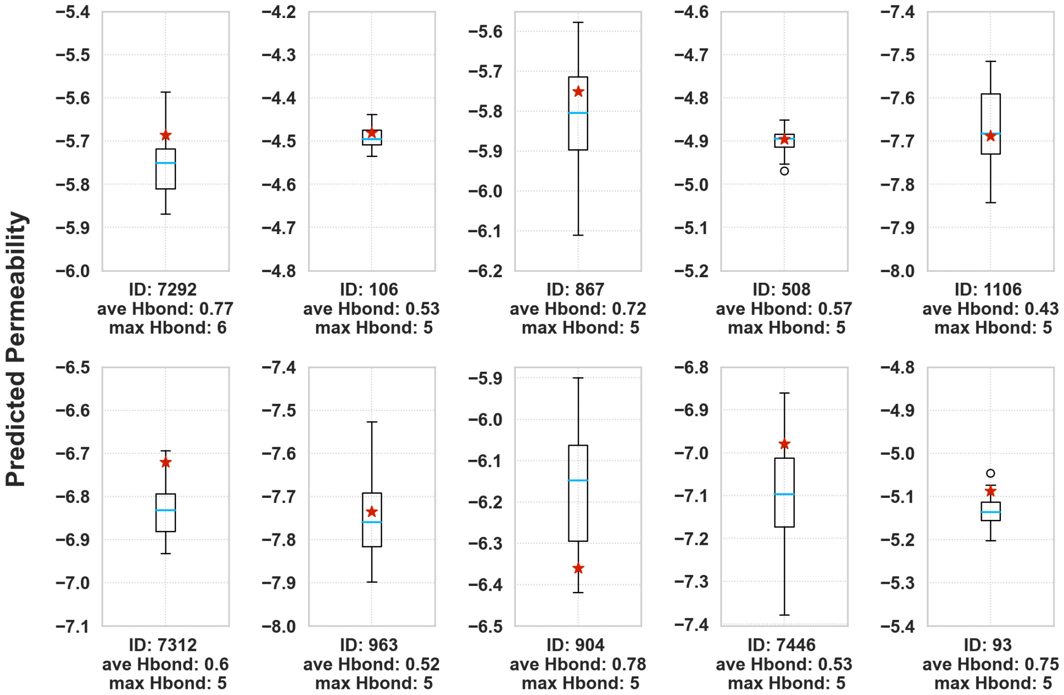

Figure S7: CycPeptMP permeability prediction results for 60 conformations of the top ten peptides considered to have the most "closed-conformations" in the test set. The predicted permeability of the conformation with the largest number of intramolecular hydrogen bonds in each of the ten peptides is indicated by a red star. Cyclic peptides with a closed conformation in hydrophobic environments often exhibit enhanced permeability. We calculated the intramolecular hydrogen bonds of the peptide conformations in the test set using PyMOL software (version 2.5.0) as there is no general definition for closed-conformation, and many studies have discussed closed-conformation based on the number of intramolecular hydrogen bonds. Here, peptides with an average number of hydrogen bonds < 0.8 were selected (generally thought to take a non-closed-conformation) to compare the predicted values of closed-conformations and non-closed-conformations; among them, the predicted values of ten peptides with the highest number of hydrogen bonds (closed-conformation) are shown. For peptides other than ID 1106 and ID 904, the predicted values with the largest number of hydrogen bond conformations (red stars) were greater than the median of predicted values for all conformations (light blue lines). The predicted values for closed-conformations tended to be slightly higher than those for non-closed-conformations. Since the predicted value changes based on the conformations, the prediction accuracy may be further improved by using conformations generated by a more rigorous method such as MD rather than the relatively simple conformations generated by the RDKit used in this study.

2 Supplemental Tables

Table S1: Search range and results of the hyperparameter search for the CycPeptMP model. Hyperparameters were determined by 150 trials using Optuna software [4] based on the average RMSE of three runs.

Objective	Hyperparameter	Description	Search range	Search result
Training	n_epochs	Epoch number.	-	50
	criterion	Loss function of training.	-	Mean Squared Error (MSE)
	n_earlystop	Number of patience for early stopping.	-	5
	scheduler	Adjustment scheduler of learning rate.	-	NoamLR
	warmup_epochs	Warming up epochs of NoamLR.	-	10 (20% of n_epochs)
	init_lr	Initial learning rate of Noam LR.	-	1e-4
	max_lr	Maximal learning rate of Noam LR.	-	1e-3
	final_lr	Final learning rate of Noam LR.	-	1e-5
	batch_size	Batch size.	64, 128, 256	256
	optimizer	Type of optimizer.	AdamW, NAdam, Radam	AdamW
	weight_deacy	Rate of L2 regularization.	5e-6, 1e-5, 5e-5, ⋯, 5e-2, 1e-1	0.1
Fusion model	d_linear	Dimension of linear layers.	64, 128, 256, 512	512
	d_subout	Dimension of sub-model output.	16, 32, 64	64
	ac	Activation function.	ReLU, LeakyReLU, SiLU, GELU	LeakyReLU
Atom model	n_encoders	Number of encoders.	1, 2, 3, 4, 5, 6	2
	dropout	Dropout rate.	0.0, 0.05, 0.10, ⋯, 0.3	0.2
	n_head	Head number of multi-head attention.	4, 8, 16, 32	16
	d_model	Dimension of encoder input.	32, 64, 128, 256	32
	d_feedforward	Dimension of feedforward network.	64, 128, 256, 512	512
	n_linears	Number of linear layers follows encoders.	1, 2	1
	λ_g	Weight of concatenating $Graph$ and $Conf$ blocks.	0.1, 0.2, ⋯, 0.9	0.9
Monomer model	n_conv	Number of convolutional layers.	1, 2, 3, 4, 5, 6	6
	conv_type	Type of convolutional layers.	1D-CNN, CyclicConv	1D-CNN
	padding	Padding size of convolutional layers.	-	1 (1D-CNN), 0 (CyclicConv)
	d_conv	Dimension of each convolutional layer.	32, 64, 128, 256	[128, 128, 32, 256, 256, 64]
	pooling	Type of pooling layer.	Max, Ave	Ave
	n_linears	Number of linear layers follows convolutional layers.	1, 2	1
Peptide model	n_mlps	Number of multi layer perceptron.	1, 2, 3, 4, 5, 6	1
	dropout	Dropout rate.	0.0, 0.05, 0.10, ⋯, 0.3	0.25
	d_mlp	Dimension of multi layer perceptron.	64, 128, 256, 512	256
Shared layer	n_shared	Number of linear layers of shared layer.	1, 2, 3	2

Table S2: Search range and results of the hyperparameter search (grid search) for the RF and SVM models.

Objective	Hyperparameter	Description	Search range	Search result
RF model	n_estimators	Number of decision trees.	50, 100, 200, 300, 500, 750, 1000	750
	max_depth	Maximum depth of each decision tree.	None, 2, 5, 10, 20, 30	20
SVM model	kernel	Kernel function.	-	Gaussian kernel (rbf)
	C	Penalty parameter.	2^{-3}, 2^{-2}, 2^{-1}, 2^{0}, 2^{1}, 2^{2}, 2^{3}, 2^{4}, 2^{5}	8 (SVM-2D), 4 (SVM-2D3D)
	γ	Kernel coefficient of Gaussian kernel.	2^{-6}, 2^{-5}, 2^{-4}, 2^{-3}, 2^{-2}, 2^{-1}, 2^{0}	2^{-1} (SVM-2D), 2^{-4} (SVM-2D3D)

Table S3: Search range and results of the hyperparameter search for the four DL-based baseline models. Hyperparameters were determined by 50 trials using Optuna software [4] based on the average RMSE of three runs.

Objective	Hyper-parameter	Description	Search range (* is the value used in the original paper)	Search result
MAT	batch_size	Batch size.	16, 32, 64, 128, 256*	64
	optimizer	Type of optimizer.	Adam*, AdamW, NAdam, RAdam	AdamW
	weight_deacy	Rate of L2 regularization.	5e-6, 1e-5, 5e-5, ⋯, 5e-2, 1e-1	5e-5
	d_model	Dimension of model.	64, 128, 256, 512, 1024*, 2048	128
	N	Number of encoder module repeats.	1, 2, 4, 6, 8*	8
	h	Number of molecule self-attention heads.	2, 4, 8, 16*, 32	32
	N_dense	Number of dense layers in the FFN.	1*, 2, 3, 4	3
	λ_att	Self-attention weight.	0, 0.1, 0.2, 0.3, 0.4, 0.5, 0.6, 0.7, 0.8, 0.9, 1	0.1
	λ_dist	Distance weight.	0, 0.1, 0.2, 0.3, 0.4, 0.5, 0.6, 0.7, 0.8, 0.9, 1	0.2
	distance_matrix_kernel	Function used to transform distance matrix.	exp*, softmax	exp
	dropout	Dropout rate.	0*, 0.1, 0.2, 0.3	0
	aggregation_type	Type of global pooling.	mean*, add	mean
SAT	batch_size	Batch size.	16, 32, 64	32
	optimizer	Type of optimizer.	Adam*, AdamW, NAdam, RAdam	AdamW
	weight_deacy	Rate of L2 regularization.	5e-6, 1e-5*, 5e-5, ⋯, 5e-2, 1e-1	1e-4
	abs_pe_dim	Dimension of absolute positional encoding.	3, 7, 10, 20*, 30	20
	k_hop	Size of subtree.	1, 2, 3*, 4, 5	4
	d_model	Dimension of model.	32, 64*, 128, 256	64
	dim_feedforward	Dimension of feedforward network.	64, 128*, 256, 512	512
	dropout	Dropout rate.	0, 0.1, 0.2, 0.3*, 0.4	0
	num_head	Head number of multi-head attention.	4, 8*, 16, 32	16
	num_layers	Number of encoders.	1, 2, 3, 4, 5, 6*	2
	norm	Type of normalization.	batch norm*, layer norm	batch norm
	gnn_type	Type of GNN-based subtree extractor.	Graph, SAGE, GCN, GIN, GINE, PNA, PNA2*, PNA3, MPNN	Graph
	global_pool	Type of global pooling.	mean*, add	mean
PharmHGT	batch_size	Batch size.	32, 64*, 128, 256	32
	optimizer	Type of optimizer.	Adam*, AdamW, NAdam, RAdam	AdamW
	weight_deacy	Rate of L2 regularization.	5e-6, 1e-5, 5e-5, ⋯, 5e-2, 1e-1	1e-3
	act	Activation function.	ReLU*, LeakyReLU, SiLU, GELU	GELU
	hid_dim	Dimension of model.	60, 120, 180, 300*, 420, 540	420
	depth	Depth of message passing.	1, 2, 3*, 4, 5, 6	2
FinGAT	batch_size	Batch size.	32*, 64, 128, 256	32
	optimizer	Type of optimizer.	Adam*, AdamW, NAdam, RAdam	Adam
	weight_deacy	Rate of L2 regularization.	5e-6, 1e-5, 5e-5, ⋯, 1e-3*, ⋯, 5e-2, 1e-1	5e-5
	ac	Activation function.	ReLU*, LeakyReLU, SiLU, GELU	SiLU
	hidden_gat	Dimension of graph attention network.	50*, 100, 150, 200, 300, 500	100
	in_head	Head number of multi-head attention.	3, 4, 5*, 6, 7, 8	8
	global_pool	Type of global pooling.	mean*, max, add	max
	hidden_linear_1	Dimension of the 1st linear layer.	10, 25, 50, 100*, 150, 200	150
	hidden_linear_2	Dimension of the 2nd linear layer.	10, 25*, 50, 100, 150, 200	200
	hidden_linear_3	Dimension of the 3rd linear layer.	10*, 25, 50, 100, 150, 200	50

Table S4: Comparing the performance between seven baseline methods and CycPeptMP using the validation set; the metrics are averaged for three runs and the best result for each metric is indicated in bold.

Metrics	RF	SVM-2D	SVM-2D3D	MAT	SAT	PharmHGT	FinGAT	CycPeptMP
MAE	0.410 ± 0.010	0.401 ± 0.012	0.392 ± 0.007	0.432 ± 0.015	0.461 ± 0.033	0.400 ± 0.014	0.394 ± 0.020	**0.355 ± 0.006**
MSE	0.328 ± 0.025	0.351 ± 0.020	0.336 ± 0.015	0.352 ± 0.008	0.388 ± 0.045	0.311 ± 0.019	0.303 ± 0.024	**0.268 ± 0.021**
R	0.716 ± 0.022	0.700 ± 0.009	0.713 ± 0.021	0.698 ± 0.022	0.651 ± 0.065	0.735 ± 0.037	0.742 ± 0.022	**0.776 ± 0.019**
R^2	0.511 ± 0.033	0.477 ± 0.011	0.498 ± 0.029	0.474 ± 0.036	0.416 ± 0.095	0.533 ± 0.056	0.547 ± 0.035	**0.600 ± 0.027**

Table S5: Prediction performance between seven baseline methods and CycPeptMP (models were trained with PAMPA) for Caco2 (378 peptides), MDCK (17 peptides), and RRCK (53 peptides) recorded in CycPeptMPDB (the duplicate peptides between each assay and PAMPA were deleted). The metrics are averaged for three runs and the best result for each metric is indicated in bold. No model could predict these assays (Caco2: MAE=0.766 to 1.18, MDCK: MAE=0.706 to 1.048, RRCK: MAE=0.567 to 0.683).

Set	Metrics	RF	SVM-2D	SVM-2D3D	MAT	SAT	PharmHGT	FinGAT	CycPeptMP
Caco2	MAE	1.124 ± 0.006	0.784 ± 0.007	**0.766 ± 0.007**	0.958 ± 0.013	1.074 ± 0.133	0.810 ± 0.131	1.180 ± 0.051	1.148 ± 0.113
	R	0.181 ± 0.002	0.279 ± 0.016	**0.290 ± 0.004**	0.175 ± 0.043	0.107 ± 0.023	0.195 ± 0.015	0.128 ± 0.040	0.209 ± 0.064
MDCK	MAE	0.913 ± 0.016	**0.706 ± 0.022**	0.778 ± 0.005	0.927 ± 0.036	0.893 ± 0.087	1.048 ± 0.093	0.929 ± 0.101	0.821 ± 0.009
	R	0.283 ± 0.021	0.377 ± 0.072	0.618 ± 0.011	0.415 ± 0.107	0.388 ± 0.144	**0.675 ± 0.037**	0.358 ± 0.080	0.570 ± 0.044
RRCK	MAE	0.683 ± 0.026	0.662 ± 0.007	0.598 ± 0.005	0.617 ± 0.062	0.650 ± 0.033	**0.567 ± 0.110**	0.682 ± 0.024	0.678 ± 0.041
	R	-0.044 ± 0.045	0.245 ± 0.006	**0.291 ± 0.016**	0.078 ± 0.066	0.242 ± 0.082	0.160 ± 0.119	-0.057 ± 0.010	-0.181 ± 0.027

Table S6: Prediction performance of CycPeptMP using 3D conformations of the test set regenerated by RDKit (five times with different seeds). The metrics are averages of three runs. The prediction accuracy had a minor change (MAE=0.448 to 0.456) using only one conformation per peptide (no augmentation). The prediction accuracy did not change when using 60 times augmentation (MAE=0.355).

Augmentation	Conformation trial	MAE	MSE	R	R^2
1 (No Augmentation)	t0 (original conformations)	0.456 ± 0.026	0.373 ± 0.036	0.823 ± 0.018	0.664 ± 0.033
	t1	0.448 ± 0.026	0.361 ± 0.034	0.827 ± 0.017	0.674 ± 0.031
	t2	0.452 ± 0.026	0.365 ± 0.034	0.825 ± 0.017	0.671 ± 0.031
	t3	0.449 ± 0.026	0.359 ± 0.033	0.828 ± 0.016	0.676 ± 0.030
	t4	0.450 ± 0.025	0.364 ± 0.034	0.825 ± 0.017	0.672 ± 0.030
	t5	0.449 ± 0.025	0.361 ± 0.033	0.827 ± 0.017	0.674 ± 0.030
60 (CycPeptMP)	t0 (original conformations)	0.355 ± 0.007	0.253 ± 0.013	0.883 ± 0.003	0.772 ± 0.011
	t1	0.355 ± 0.006	0.253 ± 0.012	0.882 ± 0.003	0.772 ± 0.011
	t2	0.355 ± 0.006	0.253 ± 0.012	0.882 ± 0.003	0.771 ± 0.011
	t3	0.355 ± 0.006	0.253 ± 0.012	0.882 ± 0.003	0.772 ± 0.011
	t4	0.355 ± 0.006	0.253 ± 0.012	0.882 ± 0.003	0.772 ± 0.011
	t5	0.355 ± 0.006	0.253 ± 0.012	0.882 ± 0.003	0.772 ± 0.011

Table S7: Peptides used in comparison with the MD-based method. The 23 peptides are included in the validation and test sets of this study. The AlogP and MD predicted value ($logP_{ISMD\_mod}$) are reported by Sugita et al [6].

Source	CycPeptMPDB ID	Compound Name	AlogP	Set in CycPeptMP	Experimental Value	MD Predicted Value	CycPeptMP Predicted Value
2013_CHUGAI	536	DP-528	0.73	Test	-4.89	-4.91	-4.76
2013_CHUGAI	538	DP-530	1.2	Test	-4.96	-5.5	-4.87
2013_CHUGAI	677	DP-712	0.78	Test	-4.92	-5.08	-4.93
2016_Furukawa	1134	1.1-01	0.12	Valid-1	-7.13	-5.06	-6.98
2016_Furukawa	1146	1.1-13	3.06	Valid-2	-4.99	-4.71	-5.10
2016_Furukawa	1151	1.1-18	2.23	Valid-1	-6.14	-4.19	-6.04
2016_Furukawa	1200	1.2-27	3.11	Valid-2	-5.34	-4.34	-5.36
2016_Furukawa	1231	1.3-18	3.08	Valid-2	-5.29	-4.3	-5.23
2016_Furukawa	1240	1.3-27	3.42	Valid-2	-5.65	-4	-5.57
2016_Furukawa	1299	1.5-06	0.83	Valid-3	-6.64	-6.81	-6.75
2016_Furukawa	1308	1.5-15	3.15	Valid-3	-5.18	-4.86	-5.34
2016_Furukawa	1311	1.5-18	1.71	Test	-6.58	-5.85	-6.80
2016_Furukawa	1345	1.6-12	1.86	Valid-3	-6.44	-4.57	-6.29
2016_Furukawa	1371	1.6-38	6.24	Valid-1	-8	-4.66	-8.00
2016_Furukawa	1388	1.7-15	6.14	Valid-1	-8	-4.24	-7.75
2016_Furukawa	1409	1.7-36	5.93	Valid-2	-7.62	-4.81	-7.66
2016_Furukawa	1424	1.8-11	2.26	Valid-1	-6.23	-5.08	-6.20
2016_Furukawa	1425	1.8-12	0.97	Test	-7.04	-4.8	-6.87
2016_Furukawa	1449	1.8-36	4.47	Test	-7.42	-4.54	-7.29
2016_Furukawa	1451	1.8-38	5.35	Valid-3	-8	-4.32	-7.99
2016_Furukawa	1454	1.9-01	1.54	Test	-5.52	-4.51	-5.66
2016_Furukawa	1471	1.9-18	3.66	Valid-2	-5.16	-5.9	-5.28
2016_Furukawa	1489	1.9-36	4.89	Valid-3	-6.93	-5.31	-6.74

References

[1] Chemical Computing Group Inc. (2019). Molecular Operating Environment (MOE), 2019.01. Montreal, QC, Canada.

[2] Moriwaki, H., Tian, Y., Kawashita, N., and Takagi, T. (2018). Mordred: a molecular descriptor calculator. *J. Cheminform.*, **10**(1), 1–14.

[3] Greg, L. (2022). RDKit: Open-source cheminformatics. https://www.rdkit.org.

[4] Akiba, T., Sano, S., Yanase, T., Ohta, T., and Koyama, M. (2019). Optuna: A next-generation hyperparameter optimization framework. In *Proceedings of the 25th ACM SIGKDD International Conference On Knowledge Discovery & Data Mining*, 2623–2631.

[5] Hutter, F., Hoos, H., and Leyton-Brown, K. (2014). An efficient approach for assessing hyperparameter importance. In *Proceedings of the 31st International Conference on Machine Learning*, 754–762.

[6] Sugita, M., Sugiyama, S., Fujie, T., Yoshikawa, Y., Yanagisawa, K., Ohue, M., and Akiyama, Y. (2021). Large-scale membrane permeability prediction of cyclic peptides crossing a lipid bilayer based on enhanced sampling molecular dynamics simulations. *J. Chem. Inf. Model.*, **61**(7), 3681–3695.
